# Retroodontoid Pseudotumor Related to Development of Myelopathy Secondary to Atlantoaxial Instability on Os Odontoideum

**DOI:** 10.1155/2018/1658129

**Published:** 2018-09-30

**Authors:** M. Hamard, S. P. Martin, S. Boudabbous

**Affiliations:** Department of Imaging and Medical Information Sciences, Division of Radiology, Geneva University Hospitals, Geneva, Switzerland

## Abstract

Retroodontoid pseudotumor (ROP) is a nonneoplasic lesion of unknown etiology, commonly associated with inflammatory conditions, and the term of pannus is usually used. Less frequently, ROP formation can develop with other noninflammatory entities, with atlantoaxial instability as most accepted pathophysiological mechanism for posttraumatic or degenerative ROP. As it can clinically and radiologically mimic a malignant tumor, it is paramount for the radiologist to know this entity. Magnetic resonance imaging is the modality of choice to reveal the possible severe complication of ROP in the form of a compressive myelopathy of the upper cervical cord. The purpose of the surgical treatment is the regression or complete disappearance of ROP, with posterior decompression by laminectomy and posterior C1-C2 or occipitocervical fixation. We present the case of an elderly patient with retroodontoid soft tissue mass secondary to a chronic atlantoaxial instability on os odontoideum, an extremely rare cause of ROP. The patient developed a posttraumatic cervical myelopathy related to the decompensation of this C1-C2 instability responsible for the formation of a compressive ROP. We will overview the retroodontoid pseudotumor and its differential diagnosis.

## 1. Introduction

Retroodontoid pseudotumor (ROP) is an entity that can mimic malignant tumors and is from uncertain etiology. Some consider it as a low-grade fibrosarcoma. ROP are mainly related to infectious processes and less frequently to inflammatory disorders. ROP might have neurological complications due to mass effect on the spinal cord.

## 2. Case Description

A 77-year-old female patient was admitted in our institution following a ground-level fall due to relatively sudden grade 3-4 right hemiparesis with lower limb predominance.

This patient was not known for any systemic disease, no rheumatoid arthritis, or other joint-related generalized disease.

**Figure 1:**
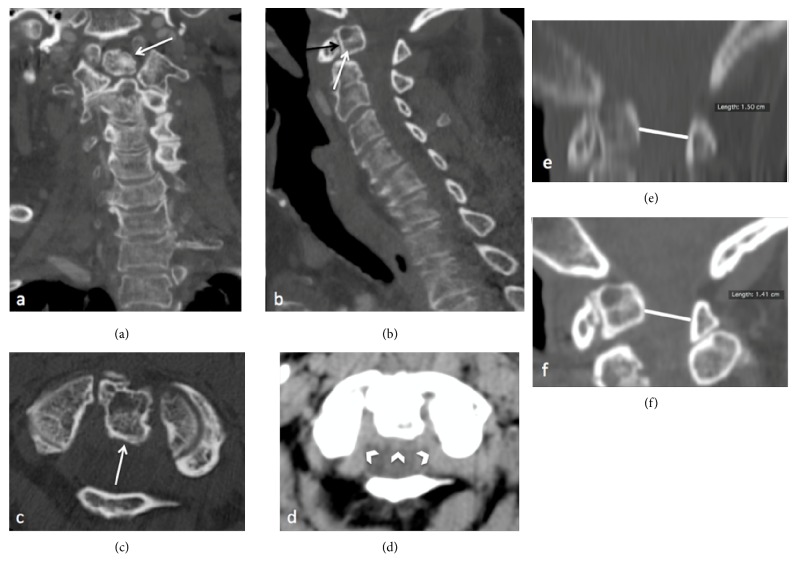
Angiography brain CT obtained to investigate the sudden right hemiparesis of the patient, showing a well-corticated bone fragment located superiorly of odontoid process in coronal reconstruction (a, white arrow) and in axial plane (c, white arrow) in bone window. This fragment seems an os odontoideum, with a pseudoarthrosis of a fracture of the dens as main differential diagnosis. In sagittal plane with bone window, it is associated with an atlantoaxial subluxation at this level (anterior arch of C1 above C2 body), with a narrowing of the space available for spinal cord between the previous 2006 CT exam (e, white line measuring 1.5 cm) and recent 2014 CT exam (f, white line measuring 1.4 cm) in bone window. There is no enlargement of the space between anterior surface of os odontoideum and the anterior arch of C1 on sagittal plane (b, black arrow). We can already see a pseudomass hyperattenuated but without enhancement in the late phase, posteriorly of os odontoideum in the cervical spinal canal in axial plane and soft tissue window (d, white arrowheads), compatible with a retroodontoid pseudotumor in ROP.

An initial enhanced CT was performed for the suspicion of an ischemic stroke. The exam revealed a smooth and well-corticated bone ossicle measuring 14 mm and located superiorly to the odontoid process corresponding to an os odontoideum (Figures 1(a)–1(c)). The ossicle was associated with an atlantoaxial subluxation and with the posterior wall of 14 mm on spinal canal (Figures 1(e) and 1(f), white lines) that has increased since a previous CT 8 years ago. The late enhanced phase showed an intracanal hyperattenuated but no enhancing pseudomass situated just posterior to the ossicle (Figure 1(d)).

**Figure 2:**
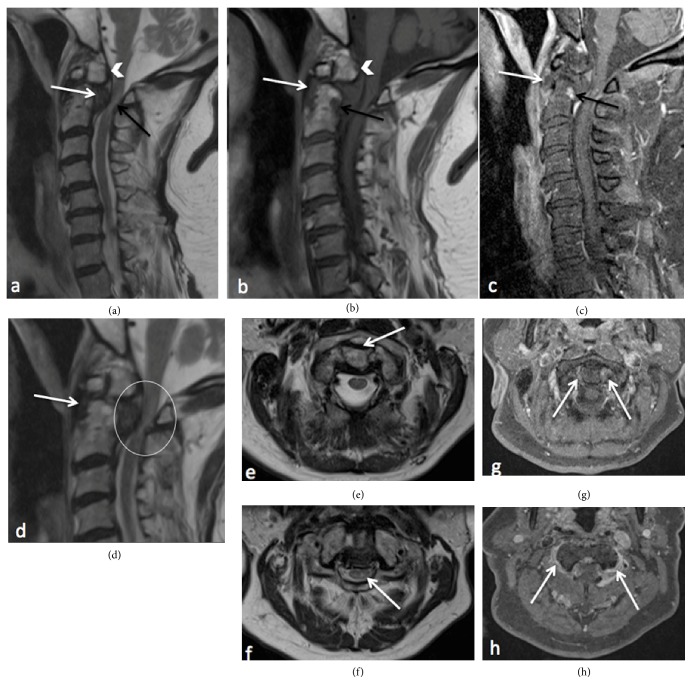
Complementary cervical enhanced MRI with Gadolinium administration, acquired to investigate the cervical spinal cord compression, showing the ROP as low signal intensity on both T1w and T2w images in sagittal planes surrounding the body of C2 and this ROP (a,b,d, white arrows). It appears without enhancement on T1w fat sat images after gadolinium administration in sagittal (c, white arrow) and axial (g, white arrows) planes. We can see some enhancement on axial T1w fat sat after contrast administration around interfacetar articulations of C1-C2 (h, white arrows), probably from degenerative origin. A sub centimetric geode in the basis of C2 shows low signal on T1w image in sagittal plane (b, black arrow) with an enhancement after contrast administration in axial plane (c, black arrow). The T2w image in sagittal plane reveals an area of high intensity in the intramedullary regions of C1 and C2 (d, white circle), due to compression by the ROP, but without diastasis between anterior arch of C1 and the dens (e, white arrow).

A complementary cervical enhanced MRI with administration of Gadolinium confirmed a well-corticated ossicle and demonstrated a tissular retroodontoid process (Figures 2(a) and 2(b)). The tissue component showed a low signal on T1- and T2-weighted images and no enhancement (Figure 2(g)), compatible with a ROP. The main diagnosis was a noninflammatory ROP developed on atlantoaxial instability, secondary to an os odontoideum. The main differential diagnosis was pseudoarthrosis of an old fracture of the dens of axis. Inflammatory arthritis such as gout, rheumatoid, or psoriatic arthritis was suggested as differential diagnosis, but less likely because of the negative history of those diseases.

There was no enlargement of space between this os odontoideum and the anterior arch of C1 (Figure 2(e)). A subcentimetric geode, in low signal on T1-weighted images and enhancement after contrast administration, was seen in the posterior dens basis of C2 (Figures 2(b) and 2(c)). The pseudotumor indenting into cervicomedullary cord and resulting in cord compression is shown (Figure 2(a)), with cervical myelopathy seen in high signal on T2-weighted images in sagittal plane (Figure 2(d)).

**Figure 3:**
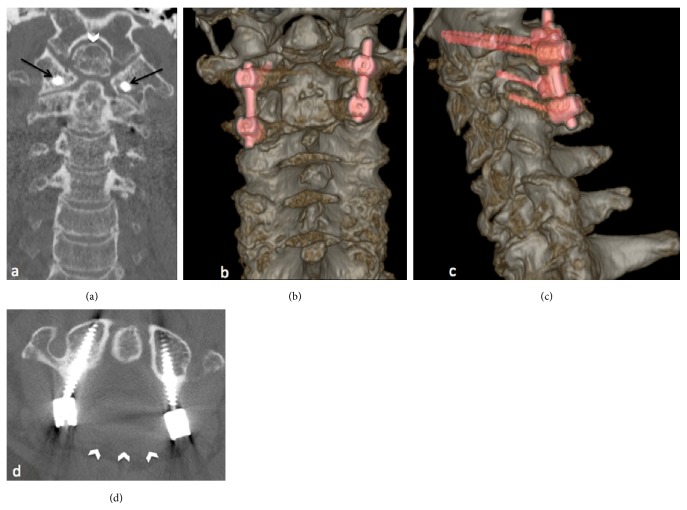
Unenhanced CT after posterior C1–C2 fixation. We see in coronal plane with bone window the transarticular C1-C2 screws (a, black arrows) and on volume rendering in coronal and sagittal reconstructions (b,c, the screws are highlighting). Spinal canal decompression by laminectomy is also made, seen in axial plane with bone window (d, white arrowheads) showing the absence of posterior arch of C1 (in comparison to image c of Figure 1). The intracanal ROP is not removed but regressed in size compared to preoperative CT.

**Figure 4:**
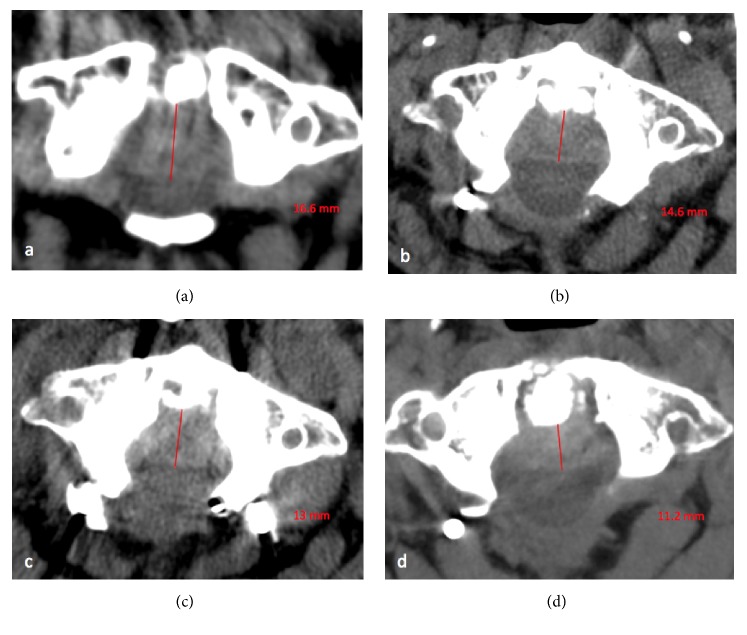
Comparison of the size of ROP on preoperative CT and postoperative CT with soft tissue window and in axial plane. We see the regression of size of the ROP between preoperative CT (a) with thickness of 16.6 mm under the os odontoideum and the postoperative CT just after surgical management (b) with thickness of 14.6 mm, at 4 months with thickness of 13 mm and at 6 months with thickness of 11.2 mm after surgery.

Given the severity of the radiological findings and the clinical impact due to spinal cord compression and the life-threatening risk, patient was treated with cervical posterior screw fixation and a decompressive laminectomy at C1-C2 level (Figures 3(a)–3(d)). Unenhanced CT after posterior C1–C2 shows posterior cervical fixation and spinal canal decompression by laminectomy. The intracanal ROP is not removed but regressed in size in each postoperative CT compared to preoperative CT (Figures 4(a)–4(d)).

**Figure 5:**
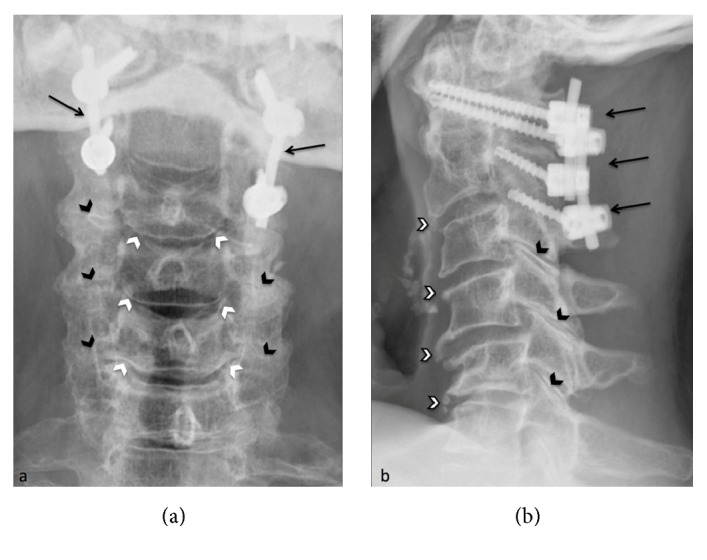
Postoperative X-rays of the cervical spine are also acquired for the follow-up. We see the posterior C1–C2 fixation with transarticular screws (a,b, black arrows). These X-rays show better degenerative changes of the cervical spine; disco-uncarthrosis (a, white arrowheads), interarticular posterior staggered arthrosis (a, b, black arrowheads), and anterior marginal osteophytosis (b, black-framed white arrowheads).

The patient showed a progressive improvement of her neurological recovery, with complete neurological recovery 6 months after surgery. In the postoperative follow-up, entire cervical spine showed marked degenerative changes, best viewed on postoperative cervical spine X-rays (Figures 5(a) and 5(b)) as disco-uncarthrosis (white arrowheads), interarticular posterior arthrosis (black arrowheads), and anterior marginal osteophytosis (black-framed white arrowheads).

## 3. Discussion

The etiology of retroodontoid pseudotumor (ROP), which can mimic malignant tumors, is unknown. Some authors believe that this entity is a low-grade fibrosarcoma [1]. When pseudotumors are related to an infectious process, the more frequent encountered organisms are mycobacteria, mycoplasma, Epstein-Barr virus, actinomycetes, and nocardiae. The noninfectious inflammatory ROP, also named pannus or phantom tumors [2], is a rare nonneoplasic reactive inflammatory granulation tissue at the craniovertebral junction typically arising from the synovium around the dens and usually associated with rheumatoid arthritis [3–5], psoriatic arthritis [6], or gout [7]. Less frequently, the noninflammatory and noninfectious entities with predisposing atlantoaxial instability (AAI) can be associated with a ROP, as os odontoideum, posttraumatic pseudoarthrosis of the odontoid fracture, and degenerative retroodontoid cysts [8]. Other entities can mimic ROP as amyloidosis [9–11], pigmented villonodular synovitis [12], epidural lipomatosis [13], and idiopathic skeletal hyperostosis [14, 15] (Table 1).

In 2004, Goel A. et al. identified that ROP indicated atlantoaxial instability (AAI) [2]. The chronic mechanical stress exerted by the AAI induces repeated tears and transverse ligamentous hypertrophy with formation of reactive fibrous granulation tissue. Patients are usually asymptomatic or can report chronic cervical pain. In more advanced and severe stage, ROP could cause cervical spinal cord compression with neurological symptoms. Occasionally, an added event can decompensate the clinical condition, like ground-level fall in our case. Pathophysiologically, noninflammatory ROP consists of fibrocartilaginous tissue between the odontoid process and the anterior arch of the C1 or between the dens and the transversal ligament [2, 16]. Thus, despite the lack of histological findings, the clinical and radiological features of our case are consistent with a noninflammatory cause. Tanaka et al. have classified ROP into 3 types, according to the etiology and MRI findings. Type 1 is the classical and common ROP, caused by AAI, and is mainly associated with rheumatoid arthritis. Type 2 shows similar MRI findings to type 1, but it is associated with spondylosis or ankylosis of the cervical segment and ossification of the anterior longitudinal ligament. In type 3, ROP is sustained by a C2-C3 disc herniation, which penetrates the posterior longitudinal ligament and migrates upward to the retroodontoid space [17].

X-rays analysis allows estimating of the atlantoaxial instability, usually with dynamic study in flexion and extension. The radiographic characteristics as ossification of the anterior longitudinal ligament and ankylosis of the adjacent segments seem to be risk factors for the formation of a ROP [18]. Distance from the posterior border of the odontoid process to the anterior border of the posterior arch of C1 can also be assessed in order to estimate the space available for spinal cord (SAC). A typical MRI finding of noninflammatory ROP is a hypo- or isointense area on T1-weighted images and an area of low or mixed intensity on T2-weighted images. ROP typically demonstrates no enhancement after gadolinium administration [19].

The surgical management of ROP aims to restore the AII and to relieve the mechanical stress at the C1-C2 junction, with spontaneous regression of ROP. Many cases report the complete disappearance of this degenerative and noninflammatory ROP following posterior C1–C2 fixation [3, 16, 20–22]. In our case, the patient showed a progressive regression of the size of the ROP on three postoperative CT scans (Figure 4) and improvement of her neurological recovery during, with complete recovery 6 months after surgery. The most used and recommended surgical procedures, with or without atlantoaxial subluxation, are the combination of spinal canal decompression by laminectomy and a posterior C1–C2 or occipitocervical fixation with transarticular screws, without removal of the intracanal ROP [2]. Tanaka et al. proposed the C1-C2 fixation with laminectomy as surgical treatment of types 1 and 2 [17].

In this case, os odontoideum is the cause for the AII with ROP. This bone ossicle is situated superiorly to the dens of axis and differs morphologically from persistent ossiculum terminale or from odontoid fracture. According to the congenital hypothesis, it corresponds to a congenital anatomic variant, due to a failure of fusion of dens center with the body of C2. The other current hypothesis supports a traumatic origin, corresponding to an odontoid process fracture type II of Anderson and Alonso classification, sustained by history of traumatism in many patients [23]. In our case, the traumatic hypothesis is more likely because of the many degenerative changes associated. On cervical spine CT, the gap between the os odontoideum and the odontoid process usually extends above the level of the superior axis facets, as in our case. Even though extremely rare, os odontoideum could be associated with atlantoaxial instability and lead to ROP [24]. There are two main types. First, the orthotopic type shows a normal position, merged with the anterior arch of C1 and a wide gap between the body of C2 and os odontoideum, as in our case. Second, the dystopic os odontoideum is cranially displaced usually fused to the basion of the clivus. This smooth small ossicle is of variable sizes, around half the size of the normal dens and associated with hypertrophied and rounded anterior arch of C1. The development of the ROP in the C2 fracture is most commonly seen in elderly population after a low-energy traumatism, with high mortality. Pseudoarthrosis of dens fracture is then usually seen in patients with history of cervical spine traumatism and osteoporosis [25].

## 4. Conclusion

We present an extremely rare case of ROP, confirmed by surgery, secondary to chronic atlantoaxial instability on os odontoideum. Our case, being the fifth reported in the literature, gives us the opportunity to remind the reader of the recent knowledge about this anatomical variant suggesting a traumatic etiology rather than a congenital source.

## Figures and Tables

**Table 1:** Differential diagnosis of ROP.

	**Clinical history**	**Symptoms**	**X-ray**	**CT scans**	**MRI**	**Diagnostic**	**Treatment and follow-up**
**ROP from atlantoaxial instability **	Elderly patients. Long term neck injury with atlantoaxial suluxation. Fracture of the odontoid process. Os odontoideum.	Usually asymptomatic. Chronic cervical pain.	Dynamic study of atlantoaxial instability. Ossification of anterior longitudinal ligament. Ankylosis.	Degenerative changes: (i) Disco-uncarthrosis. (ii) Interarticular posterior arthrosis. (iii) Osteophytosis (iv) Atlantoaxial subluxation (v) Space available for spinal cord (SAC) Ankylosis.	ROP is hypo- to iso intense on T1w and predominantly hypo intense or mixed intensity on T2w.	Fibrous or cartilaginous nature. Degenerated ligaments.	Posterior cervical fixation results in immediate postoperative neurological improvement.

**Rhumatoid or psoriasitic arthritis (RA)**	History of rhumatoid or psoriasitic arthritis.	Long standing and progressive neurologic deficits. Types 1 and 3 disappears faster than type 2.	Dynamic X-rays: severe atlantoaxial subluxation during flexion.	Erosive alterations.	Pannus predominates anteriorly to the dens, surrounds the eroded odontoid process. 3 types exists: **(1)** pannus in low signal in T1w and high signal in T2w with enhancement post contrast administration. **(2)** pseudotumour in low signal in T2w and T1w. **(3)** mixed with combination of high and low signal in T2w.		Spontaneous resolution or diminution of retrodental pannus after posterior atlantoaxial stabilisation.

**Retro-odontoid cysts **	Elderly patients with degenerative spine disease.				Cystic and hypertrophic degeneration of the transverse ligament of axis. We can differentiate this pattern from synovial cyst with echo-gradient T2-weighted images on MRI showing hemosiderin deposits and no or thin enhancement.	Transverse ligament develops granuloma formation and angiogenesis, with chronic recurrent micro hemorrhages in case of rupture, leading to cyst formation.	Excision of the cystic lesion by a trans condylar approach.

**Gouty deposits**	History of goutt (acute peripheral arthritis) Hyperuricemia.	Lombalgy > cervicalgy. All spine components can be affected. Long standing and progressive neurologic deficits.	Cord compression on myelogram.	Erosive lesion. Tohpus is hyperdense on CT, with positive diagnostic to crystal of uric acid on Dual Energy CT.	Extradural intraspinal cervical fibrous tophus, in hypo to intermediate on T1w, hypo- or hyper signal on T2w with diffuse enhancement. Ventral and lateral position to the cord. Ligament softening. Subluxation C1-C2.	Histological results showing crystals of uric acid, multinucleate giant cell and histiocyte proliferation. Macroscopically, whitish and chalky material.	Progressive clinical improvement.

**Amyloidosis**	History of long-term hemodialysis, multiple myeloma, chronic inflammatory diseases, chronic infection.	Joints pains that mimic rheumatologic disorders.	Proeminent erosive alterations.	Dorsal vertebral > lombar spine > cervical spine.	Soft tissue intra neural mass with in low intensity T1w images and hypo- or mixed intensity in T2w images. Paraspinal extension. Severe medullary cord compression is possible.	Extracellular deposition of amyloid, with normal serum amyloid associated protein. Tissue biopsy: green birefringent fibrils by polarization microscopy after staining with Congo red.	Pronostic depends of the type of amyloidosis: primary solitary amyloidosis has best recovery and of lack of recurrence with a complete resolution of amyloidoma after surgical treatment.

**Pigmented villonodular synovitis**	Uncommon Young adults Knee > hip > ankle > shoulder.	Long standing and progressive neurologic deficits.	Mass epidural between the posterior longitudinal ligament and the vertebral bodies on cervical myelogram.	No erosion.	ROP hypo intense T1.		No data.

**Epidural lipomatosis**	Diffuse overgrowth of nonencapsulated adipose tissue in the epidural space: (i) Severe obese patients. (ii) Longstanding high-dose > low dose steroid therapy (iatrogenic Cushing's syndrome).	Long standing and progressive neurologic deficits.		Fatty epidural mass with mean density of -100 to -50 HU.	Massive diffuse epidural fat compressing the entire spinal cord in hyper signal T1/T2, hypo signal in STIR. Ventral displacement of the cord. Ischemic myelopathy with central gray matter hyper signal T2w in thoracic region.		No data.

**Forestier's disease**	Older men. Diffuse idiopathic skeletal hyperostosis (DISH).	Progressive neurologic symptoms resulting from anterior cervicomedullary junction.	Massive anterior longitudinal ligament calcification with bridge on the anterior border of the thoracic and subaxial cervical spine.	Massive anterior longitudinal ligament (ALL) calcification with bridge on the anterior border of the thoracic and subaxial cervical spine.	Calcification of ALL shows hypo signal in all sequences T1/T2/STIR and no enhancement. Permits to differenciate with shiny corner in ankylosing spondylitis.	Hypertrophic degenerative cartilage.	Transoral resection of the ligamentous mass followed by occipitocervical fusion. Early postoperative neurological improvement.
